# Brain Ballet: The Choreography of Left-Right Neuroendocrine Signals in Injury

**DOI:** 10.1093/function/zqae022

**Published:** 2024-05-03

**Authors:** Marshall T Holland, Bryan Becker

**Affiliations:** Department of Neurosurgery, School of Medicine, University of Alabama at Birmingham, Birmingham, AL 35294, USA; Division of Nephrology, Department of Medicine, School of Medicine, University of Alabama at Birmingham, Birmingham, AL 35294, USA

## A Perspective on “The Left-Right Side-Specific Neuroendocrine Signaling From Injured Brain: An Organizational Principle”

A ruling dogma exists stating that motor impairment following brain injury is purely anatomical in that the loss of supraspinal input of the decussating (crossing) spinal cord tracks due to damage from traumatic brain injury (TBI), stroke, or other neurological insult results in the observed contralateral flaccidity, spasticity, and rigidity. Evidence for this dogma is based on observations of human stroke patients and in numerous animal brain injury models. In their recent article in *Function*, Watanabe and colleagues challenge the assumptions of this dogma and present compelling evidence for neuroendocrine signaling and provide exciting preclinical proof of principle experiments targeting opioid receptors to improve long-term outcomes in rodent models following unilateral brain injury (UBI).

TBI and stroke pose significant public health burdens with TBI comprising the highest yearly incidence of common neurological disorders with acute and chronic consequences. Unfortunately, current acute medical treatments are limited to medical stabilization and maneuvers to minimize secondary brain injury.^1^,^2^ The Brain Trauma Foundation Guidelines recommend specific blood pressure and cerebral perfusion pressure goals concomitant with lower intracranial pressures as first-line management.^3^ Despite multiple efforts, there have not yet been any successful trials of acute medications demonstrating long- term improvement post TBI. In fact, the CRASH study demonstrated level 1 evidence against the use of steroids in the setting of TBI despite a promising premise.^4^

Following acute stabilization, focus is directed toward long-term rehabilitation to maximize function and quality of life. Standard of care emphasizes a combination of speech, physical, and occupational rehabilitation therapy, depending on the patient’s deficits to address a patient’s long-term quality of life.^5^ Spasticity and rigidity are common consequences of traumatic brain and/or spinal cord injury and often treated with antispasmodic medications, primarily baclofen. While oral baclofen may provide some relief, higher doses cause unwanted side effects, including mental cloudiness and imbalance, that may require a higher level of intervention via placement of an intrathecal pump.^6^ Cutting edge, exploratory neurorehabilitation strategies include brain-machine interfaces and deep brain stimulation.^7^ These newer strategies, while exciting with early promise, are difficult to deploy due to the high level of expertise required. Clearly, there is a substantial need for the development of therapies, medication, or, otherwise, to improve patient outcomes in both the acute and chronic settings.

Initial evidence of a laterality-specific neurohumoral signaling pathways and contralateral limb dysfunction observed in UBI was first presented by the authors in 2020.^8^ This was demonstrated in an acute rat model preparation with complete transection of the descending neural tracts at the thoracic spinal cord at T2-T3 followed by UBI of the hindlimb sensorimotor cortex. Following UBI, this rat preparation produced contralateral hindlimb flexion, asymmetric withdrawal reflex, and asymmetric gene expression in the lumbar spinal cord. The authors discovered that hypophysectomy developed the asymmetric phenotype and serum from animals with UBI injected into normal controls produced the same asymmetric phenotype in the absence of UBI. These experiments pointed toward a neurohumoral signaling pathway independent of the descending anatomical neural pathways, and Arg-vasopressin and β-endorphin were identified as signaling molecules in left UBI. This laid the groundwork for further exploration of the neuroendocrine system that may coordinate a cascade of reactionary functions in TBI and stroke. However, these exciting experiments had major limitations that the rat preparation (T2-T3 spinal transection) did not rule out: (1) The potential contribution of the spinal somato-sympathetic nerve reflexes and (2) the timing of the brain versus spinal injury were not varied.^8^,^9^

In their current study in *Function*, Watanabe and colleagues present a series of experiments to address these major limitations and further delineate the localization and mechanisms of the neurohumoral system. Here, an updated animal model preparation with transection of the cervical spinal cord at C6-C7 is used, fully disconnecting the sympathetic nervous system from the lumbar spine. The authors repeated many of their prior experiments with randomizing the order of spinal cord transection and UBI. The authors again noted development of contralateral hindlimb asymmetry, spasticity, and increased resistance to movement with stretch (similar to findings seen in human TBI, stroke, or other neurological insult), regardless of the order of injury and abolishment of these effects with hypophysectomy with similar neurohumoral pathways and signaling genes of interest.

The authors then demonstrated the importance of the opioid receptor system as a receiver in this model. Specifically, blockade of the opioid system via administration of an opioid antagonist 24 h prior to UBI prevented development of post-brain injury asymmetry and spasticity. Interestingly, they demonstrated that specific opioid antagonists could block phenotype development unilaterally. Most excitingly, the authors obtained similar results when the antagonist was given 3-4 h after UBI. One could foresee a therapeutic administered in an emergent trauma setting. However, further work is needed given the frequent need for pain control via the opioid system in these scenarios where the direct use of opioid antagonists would not be acceptable. As the downstream mechanisms become more fully understood, our hope would be for the identification of selective targets that would result in decreased rigidity without decreased acute pain control.

In conclusion, in this set of experiments, the authors addressed prior criticisms of their rat model preparation and furthered the field’s understanding of the neurohumoral axis’ response to UBI. As noted by the authors, future studies need to investigate these findings in female rats to account for hormonal sex differences and the need to further elucidate the hypothalamic-pituitary-spinal axis response to right UBI that was not as thoroughly explored. The ability of the opioid antagonist administered 3-4 h post–brain injury to prevent the spastic phenotype represents an exciting finding and warrants further investigation. Additional understanding of the signaling pathway at the terminal receptor will be of crucial importance to provide non-opioid receptor targets that would not interfere with acute pain management. From a translational point of view, this represents a potential end goal where medications may be administered by EMS or a trauma center to improve acute and chronic outcomes from these unfortunately prevalent injuries.
